# Micrornas and Cardiovascular Diseases: From Bench to Bedside

**Published:** 2018-03-31

**Authors:** R. De Rosa, M.V. Polito, R. Benvenga, E. De Angelis, F. Piscione, G. Galasso

**Affiliations:** 1 Department of Cardiology, San Giovanni di Dio e Ruggi d’Aragona University Hospital, Salerno, IT

**Keywords:** microRNAs, atherosclerosis, heart failure, vascular remodelling, biomarkers, microRNAs-based therapeutics

## Abstract

MicroRNAs (microRNAs or miRs) are small, non-coding RNAs that control gene expression by binding to and repressing specific mRNA target and have emered as powerful regulators of many biological processes. Understanding miRNAs-biology and functions may be pivotal to get a better insight into pathophysiological mechanisms responsible for a large number of morbid conditions and may lay the foundations for the development of novel therapeutic interventions. Moreover, besides their intracellular functions, miRs are present in the human circulation in a remarkably stable cell-free form, and their plasmatic levels have been proposed as biomarkers for several pathological conditions. The present review aims to summarize the current evidences with regard to biological role of miRNAs in cardiovascular system and their involvement in the pathogenesis of cardiovascular diseases including atherosclerosis, heart failure and pathological heart and vascular remodelling and to highlight their potential use as novel biomarkers and as therapeutic targets in cardiac and vascular diseases.

## Introduction

Protein-coding genes represent only 1.1% of the human genome[1]. However, more than 70% of the human genome is transcribed giving rise to non-coding ribonucleic acids (RNAs), which are not converted into proteins but play important roles in regulation of pathophysiological processes and tissue homeostasis. Several classes of non-coding RNAs have been recognized, including microRNAs (miRNAs or miRs), long non-coding RNAs (lncRNAs) and circular RNAs (circRNAs). MicroRNAs are short (17 to 25 nucleotides), lncRNAs are composed by longer (>200 nucleotides) sequences, whereas circRNAs are generated by back-splicing reactions[2]*.* Among these latters, microRNAs are currently the subcategory of non-coding RNAs with the largest amount of evidences, in both experimental and clinical setting. Indeed, since their discovery in 1993 in Caenorhabditis elegans[3], miRNAs have emerged as key modulators of complex biological processes, including those involved in many cardiovascular diseases[4, 5]; overall, it has been estimated that between 30 and 50% of all coding genes are regulated by miRNAs. Moreover, it has been demonstrated that miRNAs are present in the human circulation in a remarkably cell-free form and that their plasmatic levels are differently regulated in many pathological conditions, thus opening attractive possibilities for use of circulating miRs as disease biomarkers[6]. Lastly, the concept of miR-based therapies is currently developing; the first miR-targeted drug has been developed in 2008 for treatment of hepatitis C and is currently tested in a phase 2b clinical trial; in cardiovascular field, promising perspectives are arising from preclinical studies involving a wide range of cardiovascular disorders, although several concerns remain to be overcome. Aim of the present review is to provide an overview of microRNAs-biology and of the most relevant experimental evidence underlying their involvement in cardiovascular diseases, summarize the current evidence of their potential role as novel disease biomarkers and discuss potentialities and limitations of miR-therapeutics in different cardiac and vascular pathologic conditions.

## Biology of microRNAs

The transcription of the primary miRNA (pri-miRNA) from the genome is mediated by the nuclear RNA polymerase II[7]. The pri-miRNA is processed into a precursor miRNA (pre-miRNA), composed from 60–70 nucleotides, by the enzyme complex of Drosha-Dgcr8; the pre-miRNA is thereafter transported to the cytoplasm by the GTP-dependent nuclear export factor Exportin-5 and here it is cleaved by the RNAse III enzyme complex into the mature duplex miRNA. Of this latter, one strand (the mature microRNA) is incorporated into the miRNA-induced silencing complex (miRISC), while the other strand (passenger strand) is usually degraded. More recently, non-canonical pathways for miRNA-biogenesis, alternatively including Drosha- or Dicer-independent mechanisms, have emerged[8]. The main biological function of miRNAs is to regulate gene expression at the post-transcriptional level, by binding to specific mRNAs; depending on the degree of complementarity, a specific miRNA may either induce the degradation of the target mRNA or, most frequently, prevent its translation into proteins[7]. The regulatory function of miRNAs is powerful and fine-regulated. Indeed, because of the partial complementarity, a single miR can modulate the expression of several genes; moreover, miRs with partial overlapping of their binding sites can target the same gene-translate and can interact each other to form miRs-clusters, thus making their regulatory potential even larger.

## MicroRNAs and cardiovascular system

The crucial role of miRNAs in the cardiovascular system is supported by the finding that depletion of miRNAs-processing enzyme Dicer leads to defects in angiogenesis, vessel formation and cardiac development. Specific miRs are differently expressed in various cells and tissues composing the cardiovascular system. In detail, several miRs are expressed in endothelial cells (ECs), where they act as regulators (activators-like miR-27b, miR-130a, miR-126, or inhibitors-like miR-221 and miR-222) of angiogenesis and vasculogenesis[9, 10]; other miRs are expressed in vascular smooth muscle cells (VSMCs) and regulate their differentiation and contractile function[11, 12]; further, other miRs (like miR-1 and miR-208) are preferentially expressed in cardiomyocytes and are involved in physiological cardiac development as well as in pathological myocardial hypertrophy[13–15]. In the present review, we focused on miRs that have been demonstrated to be involved in common pathological cardiovascular conditions as atherosclerosis, vascular remodelling, coronary artery disease and heart failure.

## MicroRNAs and atherosclerosis

Atherosclerosis is a multifactorial, chronic disease whose pathophysiological mechanisms remained currently only partially understood. Pivotal processes in initiation and development of atherosclerosis included endothelial cells dysfunction, infiltration of vascular intima by inflammatory cells, lipid accumulation and vascular smooth muscle cells (VSMCs) dysregulation. In detail, endothelial dysfunction is thought to represent the initiating step in the development of an atherosclerotic plaque: following various potential form of injuries (i.e., increased vascular wall shear stress, pressure overload, increased levels of LDL or free radicals, diabetes and so on), EC function is impaired leading to increases endothelial permeability and up-regulation of leucocyte and endothelial adhesion molecules. Then, circulating leucocytes adhere to activated endothelium and migrate into the artery wall; in particular, T-lymphocytes and lipid-laden macrophages (foam cells) accumulate in the vascular wall and release proteases, cytokines, chemokynes and growth factors that perpetuate the inflammation process. In addition, vascular smooth muscle cells (VSMCs) migrate and proliferate within the developing plaque and undergo relevant phenotypic changes, switching form a synthetic to a proliferative state that has a pivotal role in the further expansion of the atherosclerotic lesion.

Specific miRNAs have been shown to play a key role in the modulation of various important processes linked to vascular and endothelial cell biology[5]. In cultured ECs, several specific miRs have been shown to stimulate or inhibit angiogenesis[16]. Importantly, miRNAs have indeed been shown to be involved in most of the steps necessary for the development of an atherosclerotic plaque. MiR-126 is one of the first miR to be identified as involved in the development of atherosclerosis. MiR-126 is highly expressed in EC and its expression is essential for vascular development[17]. Experimental studies have shown that mir-126 is key in maintaining a proliferative reserve of ECs in response to shear stress and that reduced levels of this miR reduced ECs proliferation, promoting plaque formation[18, 19]. Furthermore, miR-126 has been shown to inhibit the expression of vascular cell adhesion molecule-1 (VCAM-1), thus preventing leucocyte adherence to ECs[20]. On the contrary, miR-21 has been demonstrated to increase neointima formation and ECs-apoptosis, thus favouring atherosclerotic process. With regard to inflammation, expression of miR-155 has been shown to be induced by cytokines like TNF-α and IFN-β and such increased expression contributes to physiological expansion of granulocyte and monocyte populations during inflammation. MiR-155 is ubiquitously expressed and, in the context of an atherosclerotic lesion, it has been shown to modulate different biological processes in ECs, in foam cells and in VSMCs. On the contrary, miR-143 and miR-145 are preferentially expressed in VSMCs, where they act as regulator of their differentiation. Recently, the muscle-enriched miR-133, initially identified in cardiac and skeletal muscle, has been demonstrated to be also expressed in VSMCs, where it regulates their “phenotypic switch” (from a contractile to a synthetic status) in response to vascular injury[21].

## Coronary artery disease

Specific microRNAs and microRNAs-cluster have been shown to be modulated in the setting of both stable and unstable coronary artery disease. Circulating levels of microRNAs that are highly expressed in the myocardium (miR-1, miR-133, miR-208 and miR-499) are significantly increased in patients with ACS, and have been shown to be directly correlated with extent of myocardial damage and with patients’ prognose[22–24]. Therefore, these miRs have been proposed as novel tools to improve specificity and sensitivity of established markers of myocardial injury like high-sensitivity Troponin T (hs-Trop T) in the diagnosis of ACS. In particular, specific muscle-enriched miRs have been shown to be released very early during ACS, within 30 minutes from symptoms onset[22], and could therefore be useful in the diagnosis of ACS patients presenting early, at a time where hs-Troponin T is still negative. Moreover, detection of specific miRs linked with myocardial ischaemia could be useful in the differential diagnosis among several pathological conditions potentially associated with elevation of traditional cardiac biomarkers[25, 26]. Beyond the acute setting, several studies suggested that a number of microRNAs may be useful novel biomarkers in the diagnosis and prognosis of patients suffering from stable CAD. In detail, microRNAs highly expressed in endothelial cells (miR-126, miR17, miR92a) have been found to be significantly downregulated in patients with CAD compared with healthy controls. Moreover, three microRNAs (miR-126, miR-197 and miR-223) were able to predict subsequent AMI in the general population at 10-years follow-up[27]. Interestingly, changes in the kinetics of miR-concentrations through the transcoronary circulation have been linked with overall coronary plaque burden and morphological plaque characteristics in patients with stable CAD. In details, transcoronary concentration gradients of the anti-atherosclerotic miR-126 and miR-145 were associated with extent of coronary vulnerable plaques, suggesting that the local uptake or degradation of specific miRs may be linked with instable plaque phenotype[28].

## Pathological vascular remodelling

Pathological vascular remodelling is a widely used term to indicate a great number of dynamic processes leading to active structural changes in the vessel wall structure[29] in response to vascular injury. VSMCs and extracellular matrix (ECM) proteins are principally involved in this process. It is well known that VSMCs, unlike adult skeletal and cardiac muscle cells, retain a remarkable phenotypic plasticity and can undergo relevant changes in gene expression, contractile function, cell cycle state and production of ECM and inflammatory proteins, which represent one of the first steps in the development of an atherosclerotic lesion[30]. Recently, miR-133a has been shown to be involved in the “phenotypic switch” of VSMCs from a contractile to a proliferative state, suggesting that this miR could indeed play an important role in the development of pathological vascular remodelling and in vasculoproliferative diseases[21]. Moreover, increased cardiac release of this miR has been recently associated with higher incidence of restenosis after coronary stent-implantation[24]. Another miR that was associated with vascular remodelling is miR-29b. This miR was first described as a stress-induced miR in the heart and has emerged as novel key regulator of vascular homeostasis, moderating the expression levels of multiple gene transcripts encoding various components of extracellular matrix (ECM), like type I and type III collagen, fibrillin-1 and elastin[31]. All miR-29-family members act as endogenous inhibitors of ECM proteins expression and, consistently, their downregulation in vivo induces a fibrotic response in various organs, including heart[31], liver[32], kidney[33] and skin[34]. Similarly, in the vasculature miR-29 targets play a pivotal role in maintaining the integrity of the vessel wall. In this setting, it has been shown that miR-29b plays a pivotal role in the formation of aortic aneurysms and that its inhibition reduces aneurysm formation in different murine models[35, 36]. In the atherosclerotic coronary tree, miR-29b levels have been found to be reduced in patients with larger extent of fibrotic coronary plaques consistently with experimental studies demonstrating that a reduction of miR-29b may lead to an up-regulation of ECM proteins and, therefore, increase tissue fibrosis[28]. Lastly, miR-34 has been associated with age-related vessel degeneration; this miR is upregulated in senescent endothelial cells and VSMCs, where it targets pivotal cell cycle regulators (including SIRT1 and Bcl-2) promoting cellular death[37].

## MicroRNAs, pathological cardiac remodelling and heart failure

Alterations in microRNAs-biogenesis pathways have been demonstrated to be linked with biventricular dilatation and fibrosis, myocyte hypertrophy, foetal gene reprogramming and sudden death, implicating a pivotal role of miRNAs in cardiac remodelling and development of heart failure (HF). Cardiomyocyte-enriched miRNAs miR-1 and miR-133 have been extensively studied in this pathological setting. In detail, expression of both miRs has been found to be inversely associated with cardiac hypertrophy in animal and human specimens[13], and forced expression of miR-1 was able to revert cardiac hypertrophy in an animal model[38]. In contrast, reduced expression of cardiac-specific miR-208 has been linked with improvement of cardiac function and increased survival in a rat model of HF[38]. Interestingly, specific miRs have been shown to be involved in alterations of specific regulatory pathways in different pathogenetic and clinical settings of HF. For instance, experimental overexpression of miR-24 in ischemic myocardium has been associated with reduced infarct size, improved cardiac function and prevention of HF[39]. On the contrary, increased expression of miR-34 has been associated with pathological left ventricular remodelling after acute myocardial infarction[40], as well as with HF of non-ischemic aetiology[41]; indeed, experimental studies have demonstrated that miR-34 is induced in the ageing heart and that in vivo silencing or genetic deletion of this miR reduces age-associated cardiomyocyte cell death, contributing to the age-dependent decline in cardiac function. Mechanistically, identified miR-34 target PNUTS and Foxo3a are known to play a pivotal role in the modulation of apoptosis and myocardial cell loss, contributing to chronic myocardial damage both in ischaemic and non-ischaemic HF[37, 42, 43]. Modulation of endothelial-enriched miR-126 has also been reported in patients with HF, where they are correlated with age, LV-function, NT-proBNP levels and atrial volume[44]. Moreover, reduced miR-126 levels in circulating endothelial progenitor cells (EPCs) result in reduced cardiac repair potential, impaired EPCs-mobilization and homing in response to myocardial hypoxia and impaired angiogenesis and it is associated with poor patients’ prognosis[45, 46].

## Potential therapeutic applications

Given their key role in the regulation of a great number of biological processes involved in the disease pathogenesis, miRNAs-modulation represents a promising novel therapeutic strategy. Therapeutic application of miRs include the use of either inhibitors of miRs (antagomiRs, also named anti-miRs or blockmiRs) or overexpression of miRs by using adeno-associated viruses[47]. Currently, use of anti-miRs is preferred over that of adenovirus because of a better safety profile. AntimiRs are chemically engineered oligonucleotides developed in order to prevent the miRs-modulated posttrascriptional gene regulation[48]. AntagomiRs have a sequence that is complementary to a mRNA-sequence that serves as a binding site for microRNA; upon binding, antagomiRs sterically inhibit miRs from binding at the same site, and so prevent the miRs-induced degradation of target mRNA. The first microRNA-targeted drug (Miravirsen) has been developed in 2008 for the therapy of Hepatitis C virus (HCV) infection to prevent miR-122-mediated stability and propagation of HCV-RNA. The results of the phase 2a clinical trial of miravirsen reported a dose-dependent reduction of HCV-RNA levels after five weekly injection, that lasted up to 10 weeks after the active treatment phase, moreover showing an excellent safety profile[49]. In the cardiovascular field, antagomiRs against miR-33a and miR-33b (miRs involved in the regulation of cholesterol transport) led to sustained elevation of plasmatic high-density lipoprotein (HDL) cholesterol and reduction in very-low-density lipoprotein (VLDL) cholesterol without any adverse side effects in non-human primates[50]. Along the same line, antagomiRs against microRNA-29b have been developed and are currently tested in animals for the healing of aortic aneurysms[51]. Despite these encouraging premises, several concerns regarding the potential therapeutic use of miRs-modulators in cardiovascular system have been taken into account. In particular, it has to been underlined that the most miRs identified as potential therapeutic targets in cardiovascular diseases are ubiquitously expressed, raising concerns with regard to both efficiency of the therapy and “off-targets” systemic effects. Indeed, targeting the cardiovascular system would require higher anti-miRs doses compared with those required when antimiRs are directly delivered into the target organ, and adverse effects could occurr (i.e., nephrotoxicity). Moreover, monitoring of successful target engagement appears to be difficult in a systemic setting. Thus, strategies allowing local or cell-specific delivery of miRs and systems to monitor on-target effects could be required before miRNA-manipulations could become a reliable and feasible therapeutic intervention in the clinical practice.

## Conclusions

Continuously growing evidence shows that microRNAs play an active and pivotal role in many biological processes involved in the pathogenesis of several widespread cardiovascular diseases. Data deriving from clinical and experimental studies open attractive possibilities for use of microRNAs as diagnostic and prognostic biomarkers in the clinical practice and could lay the foundation for development of microRNAs-based therapeutic interventions.

## References

[1] Venter JC, Adams MD, Myers EW, Li PW, Mural RJ, Sutton GG (2001). The sequence of the human genome. Science.

[2] Memczak S, Jens M, Elefsinioti A, Torti F, Krueger J, Rybak A (2013). Circular RNAs are a large class of animal RNAs with regulatory potency. Nature.

[3] Lee RC, Feinbaum RL, Ambros V (1993). The C. elegans heterochronic gene lin-4 encodes small RNAs with antisense complementarity to lin-14. Cell.

[4] Condorelli G, Latronico MV, Dorn GW (2010). 2nd: microRNAs in heart disease: putative novel therapeutic targets?. Eur Heart J.

[5] Urbich C, Kuehbacher A, Dimmeler S (2008). Role of microRNAs in vascular diseases, inflammation, and angiogenesis. Cardiovasc Res.

[6] Chen X, Ba Y, Ma L, Cai X, Yin Y, Wang K (2008). Characterization of microRNAs in serum: a novel class of biomarkers for diagnosis of cancer and other diseases. Cell Res.

[7] Ambros V (2004). The functions of animal microRNAs. Nature.

[8] Yang JS, Lai EC (2011). Alternative miRNA biogenesis pathways and the interpretation of core miRNA pathway mutants. Mol Cell.

[9] Bonauer A, Carmona G, Iwasaki M, Mione M, Koyanagi M, Fischer A (2009). MicroRNA-92a controls angiogenesis and functional recovery of ischemic tissues in mice. Science (New York, NY.

[10] Suarez Y, Fernandez-Hernando C, Yu J, Gerber SA, Harrison KD, Pober JS (2008). Dicer-dependent endothelial microRNAs are necessary for postnatal angiogenesis. Proceedings of the National Academy of Sciences of the United States of America.

[11] Boettger T, Beetz N, Kostin S, Schneider J, Kruger M, Hein L (2009). Acquisition of the contractile phenotype by murine arterial smooth muscle cells depends on the Mir143/145 gene cluster. J Clin Invest.

[12] Elia L, Quintavalle M, Zhang J, Contu R, Cossu L, Latronico MV (2009). The knockout of miR-143 and -145 alters smooth muscle cell maintenance and vascular homeostasis in mice: correlates with human disease. Cell death and differentiation.

[13] Care A, Catalucci D, Felicetti F, Bonci D, Addario A, Gallo P (2007). MicroRNA-133 controls cardiac hypertrophy. Nat Med.

[14] van Rooij E, Sutherland LB, Qi X, Richardson JA, Hill J, Olson EN (2007). Control of stress-dependent cardiac growth and gene expression by a microRNA. Science (New York, NY.

[15] Zhao Y, Ransom JF, Li A, Vedantham V, von Drehle M, Muth AN (2007). Dysregulation of cardiogenesis, cardiac conduction, and cell cycle in mice lacking miRNA-1-2. Cell.

[16] Kuehbacher A, Urbich C, Zeiher AM, Dimmeler S (2007). Role of Dicer and Drosha for endothelial microRNA expression and angiogenesis. Circulation research.

[17] Fish JE, Santoro MM, Morton SU, Yu S, Yeh RF, Wythe JD (2008). miR-126 regulates angiogenic signaling and vascular integrity. Developmental cell.

[18] Schober A, Nazari-Jahantigh M, Wei Y, Bidzhekov K, Gremse F, Grommes J (2014). MicroRNA-126-5p promotes endothelial proliferation and limits atherosclerosis by suppressing Dlk1. Nat Med.

[19] Zernecke A, Bidzhekov K, Noels H, Shagdarsuren E, Gan L, Denecke B (2009). Delivery of microRNA-126 by apoptotic bodies induces CXCL12-dependent vascular protection. Sci Signal.

[20] Harris TA, Yamakuchi M, Ferlito M, Mendell JT, Lowenstein CJ (2008). MicroRNA-126 regulates endothelial expression of vascular cell adhesion molecule 1. Proc Natl Acad Sci U S A.

[21] Torella D, Iaconetti C, Catalucci D, Ellison GM, Leone A, Waring CD (2011). MicroRNA-133 controls vascular smooth muscle cell phenotypic switch in vitro and vascular remodeling in vivo. Circ Res.

[22] Rosa De S, Fichtlscherer S, Lehmann R, Assmus B, Dimmeler S, Zeiher AM: Transcoronary concentration gradients of circulating microRNAs. Circulation.

[23] Eitel I, Adams V, Dieterich P, Fuernau G, de Waha S, Desch S (2012). Relation of circulating MicroRNA-133a concentrations with myocardial damage and clinical prognosis in ST-elevation myocardial infarction. Am Heart J.

[24] Rosa De (2017). R, De Rosa S, Leistner D, Boeckel JN, Keller T, Fichtlscherer S et al: Transcoronary Concentration Gradient of microRNA-133a and Outcome in Patients With Coronary Artery Disease. Am J Cardiol.

[25] D’Alessandra Y, Devanna P, Limana F, Straino S, Di Carlo A, Brambilla PG (2010). Circulating microRNAs are new and sensitive biomarkers of myocardial infarction. Eur Heart J.

[26] Wang GK, Zhu JQ, Zhang JT, Li Q, Li Y, He J (2010). Circulating microRNA: a novel potential biomarker for early diagnosis of acute myocardial infarction in humans. Eur Heart J.

[27] Zampetaki A, Willeit P, Tilling L, Drozdov I, Prokopi M, Renard JM (2012). Prospective study on circulating MicroRNAs and risk of myocardial infarction. J Am Coll Cardiol.

[28] Leistner DM, Boeckel JN, Reis SM, Thome CE, De Rosa R, Keller T (2016). Transcoronary gradients of vascular miRNAs and coronary atherosclerotic plaque characteristics. Eur Heart J.

[29] Gibbons GH, Dzau VJ (1994). The emerging concept of vascular remodeling. N Engl J Med.

[30] Owens GK, Kumar MS, Wamhoff BR (2004). Molecular regulation of vascular smooth muscle cell differentiation in development and disease. Physiol Rev.

[31] van Rooij E, Sutherland LB, Thatcher JE, DiMaio JM, Naseem RH, Marshall WS (2008). Dysregulation of microRNAs after myocardial infarction reveals a role of miR-29 in cardiac fibrosis. Proc Natl Acad Sci U S A.

[32] Hyun J, Choi SS, Diehl AM, Jung Y (2014). Potential role of Hedgehog signaling and microRNA-29 in liver fibrosis of IKKbeta-deficient mouse. J Mol Histol.

[33] Qin W, Chung AC, Huang XR, Meng XM, Hui DS, Yu CM (2011). TGF-beta/Smad3 signaling promotes renal fibrosis by inhibiting miR-29. J Am Soc Nephrol.

[34] Maurer B, Stanczyk J, Jungel A, Akhmetshina A, Trenkmann M, Brock M (2010). MicroRNA-29, a key regulator of collagen expression in systemic sclerosis. Arthritis Rheum.

[35] Boon RA, Seeger T, Heydt S, Fischer A, Hergenreider E, Horrevoets AJ (2011). MicroRNA-29 in aortic dilation: implications for aneurysm formation. Circ Res.

[36] Maegdefessel L, Azuma J, Toh R, Merk DR, Deng A, Chin JT (2012). Inhibition of microRNA-29b reduces murine abdominal aortic aneurysm development. J Clin Invest.

[37] Boon RA, Iekushi K, Lechner S, Seeger T, Fischer A, Heydt S (2013). MicroRNA-34a regulates cardiac ageing and function. Nature.

[38] Montgomery RL, Hullinger TG, Semus HM, Dickinson BA, Seto AG, Lynch JM (2011). Therapeutic inhibition of miR-208a improves cardiac function and survival during heart failure. Circulation.

[39] Limana F, Esposito G, D’Arcangelo D, Di Carlo A, Romani S, Melillo G (2011). HMGB1 attenuates cardiac remodelling in the failing heart via enhanced cardiac regeneration and miR-206-mediated inhibition of TIMP-3. PLoS One.

[40] Lv P, Zhou M, He J, Meng W, Ma X, Dong S (2014). Circulating miR-208b and miR-34a are associated with left ventricular remodeling after acute myocardial infarction. Int J Mol Sci.

[41] De Rosa S, Eposito F, Carella C, Strangio A, Ammirati G, Sabatino J (2018). Transcoronary concentration gradients of circulating microRNAs in heart failure. Eur J Heart Fail.

[43] Galasso G, De Rosa R, Piscione F, Iaccarino G, Vosa C, Sorriento D (2010). Myocardial expression of FOXO3a-Atrogin-1 pathway in human heart failure. Eur J Heart Fail.

[44] Wei XJ, Han M, Yang FY, Wei GC, Liang ZG, Yao H (2015). Biological significance of miR-126 expression in atrial fibrillation and heart failure. Braz J Med Biol Res.

[45] Galasso G, De Rosa R, Ciccarelli M, Sorriento D, Del Giudice C, Strisciuglio T (2013). beta2-Adrenergic receptor stimulation improves endothelial progenitor cell-mediated ischemic neoangiogenesis. Circ Res.

[46] Qiang L, Hong L, Ningfu W, Huaihong C, Jing W (2013). Expression of miR-126 and miR-508-5p in endothelial progenitor cells is associated with the prognosis of chronic heart failure patients. Int J Cardiol.

[47] Thum T, Catalucci D, Bauersachs J (2008). MicroRNAs: novel regulators in cardiac development and disease. Cardiovasc Res.

[48] Krutzfeldt J, Rajewsky N, Braich R, Rajeev KG, Tuschl T, Manoharan M, Stoffel M (2005). Silencing of microRNAs in vivo with ‘antagomirs’. Nature.

[49] Janssen HL, Reesink HW, Lawitz EJ, Zeuzem S, Rodriguez-Torres M, Patel K (2013). Treatment of HCV infection by targeting microRNA. N Engl J Med.

[50] Rayner KJ, Esau CC, Hussain FN, McDaniel AL, Marshall SM, van Gils JM (2011). Inhibition of miR-33a/b in non-human primates raises plasma HDL and lowers VLDL triglycerides. Nature.

[51] Boon RA, Seeger T, Heydt S, Fischer A, Hergenreider E, Horrevoets AJ MicroRNA-29 in aortic dilation: implications for aneurysm formation. Circulation research.

